# Microstructural, Corrosion Resistance, and Tribological Properties of Al_2_O_3_ Coatings Prepared by Atmospheric Plasma Spraying

**DOI:** 10.3390/ma15249013

**Published:** 2022-12-16

**Authors:** Costică Bejinariu, Viorel Paleu, Ciprian Vasile Stamate, Ramona Cimpoeșu, Margareta Coteață, Gheorghe Bădărău, Mihai Axinte, Bogdan Istrate, Gabriel Dragos Vasilescu, Nicanor Cimpoeșu

**Affiliations:** 1 Materials Science and Engineering Faculty, Gheorghe Asachi Technical University of Iasi, 700050 Iasi, Romania; 2 Mechanical Engineering, Mechatronics and Robotics Department, Mechanical Engineering Faculty, Gheorghe Asachi Technical University of Iasi, 700050 Iași, Romania; 3 Faculty of Machine Manufacturing and Industrial Management, Gheorghe Asachi Technical University of Iasi, 700050 Iasi, Romania; 4 “INSEMEX” National Institute for Research and Development in Mine Safety and Protection to Explosion, 332029 Petrosani, Romania

**Keywords:** coatings, A_l2_O_3_, plasma spraying, SEM, EDS, AFM

## Abstract

An usual material, EN-GJL-250 cast iron, used for automotive braking systems, was covered with a ceramic material (105NS-1 aluminium oxide) using an industrial deposition system (Sulzer Metco). The main reason was to improve the corrosion and wear (friction) resistance properties of the cast-iron. Samples were prepared by mechanical grinding and sandblasting before the deposition. We applied two and four passes (around 12–15 µm by layer) each at 90° obtaining ceramic coatings of 30 respectively 60 µm. The surface of the samples (with ceramic coatings) was investigated using scanning electron microscopy (SEM), dispersive energy spectroscopy (EDS) and X-ray diffraction (XRD). Scratch and micro-hardness tests were performed using CETR-UMT-2 micro-tribometer equipment. The better corrosion resistance of the base material was obtained by applying the ceramic coating. The results present a better corrosion resistance and a higher coefficient of friction of the coated samples.

## 1. Introduction

Over the years, the field of materials destined for friction systems has evolved very much due to the increasingly needed answers to more precious operation requests [1]. The operating conditions have become more and more demanding both because of the increasing weight of the bodies involved in the braking process and of their increasing speed. The materials used for the manufacturing of a braking system make a friction couple that must ensure the transformation of the kinetic energy of the system into thermal energy by using two friction surfaces. The braking discs are parts used for slowing down or stopping a wheel from its rotational movement. These discs are made from Fe-C cast alloys, but in some cases, those that cost more, can be made using composite materials, for example, C—reinforced C or ceramic compounds [2].

A special interest is given to metallic braking discs having applications in the auto, railroad, and aeronautical fields because of the promoted prices and of the recognised already approved technologies. The geometric morphology of the discs, the width and first of all the material used for building them, bring great advantages to such equipment from the commercial perspective. Based on the thermal conductivity, special wear resistance and machinability the braking discs made from Fe-C alloys will continue to represent an important issue in what concerns the developments in the field. The analysis of the braking discs does not represent just immediate gain having applications in the automobile industry, but also an opportunity in various fields such as aeronautics and industrial at every scale [3].

Ceramic materials such as alumina (Al_2_O_3_) represent a very good solution as coatings in different industrial fields, such as aero spatial elements, building constructions, and electrical and electronic domains, mainly based on their very good properties at high temperatures such as mechanical strength, wear resistance, and in many cases corrosion-resistant properties [4,5,6,7].

For the laboratory level, there are different thermal spraying methods used (Vacuum Plasma Spraying—VPS; Combustion Flame Spraying—CFS; Two-Wire Electric Arc Spraying—TWEAS, Plasma Spraying—PS or High-Velocity oxy-fuel spraying—HVOF). Among them, Atmospheric Plasma Spraying (APS) is extensively used for the growth of ceramic coatings, oxides, or non-oxide based on its higher deposition rate and lower cost [8]. For industrial applications, using APS with a robotic arm increases the possibilities of improving the properties of already used metallic parts using ceramic coatings. Along with the high deposition rate of the process, we deal with various quality problems characteristic of APS layers, such as high porosity, low adhesion, and reduced gas jet velocity, resulting in ceramic layers with poor mechanical properties, wear resistance, or decrease in high-temperature corrosion resistance. [9].

Plasma deposition shows the advantages of a higher deposition rate, larger surfaces covered and the possibility of deposition for a wide range of materials (metallic, ceramic or polymers). For industrial applications, it is important to determine the optimum deposition parameters, which directly depend on the nature of the deposited layers and of the substrate, such as the number of deposited layers, the spraying distance, or substrate roughness.

In this paper, there are shown the experimental results obtained on samples of EN-GJL-250 cast iron covered with a ceramic coating of alumina (two and four layers deposited successively) using a plasma jet spraying method after the structural, mechanical, and chemical analysis of new materials. The subject of the paper was proposed by a private industrial company aiming for the optimisation of the APS deposition process. The results encourage the usage of APS of ceramic layer and for a satisfactory covering of metallic surface deposition of more than two layers. 

## 2. Experimental Details

The plasma jet was covered by Ar (pressure 5.2 bar and gas flow 39 NLPM) and H (pressure 3.4 bar and gas flow 6.6 NLPM). We applied two and four passes (around 12-15 µm by layer) [10]. Samples were prepared by mechanical grinding and sandblasting before the deposition. The experimental setup is presented in ref. [10] and is formed by rotational support, an automatic arm for pulverization and a holder for samples. The surface of the experimental samples (with two and four ceramic layers) was investigated using scanning electron microscopy (S.E.M.—Vega-Tescan LMHII, 25kV, high vacuum, 25.5 mm WD, W cathode), dispersive energy spectroscopy (EDX—Bruker, X-Flash detector, automatic mode), atomic force microscopy (A.F.M. EasyscanII-Nanosurf, in contact mode) and X-ray diffraction (X.R.D.—Panalytical equipment). The general surface profile of the ceramic coating was determined using a Taylor-Hobson precision profile meter and the scratch and micro-hardness tests were performed using CETR-UMT-2 micro-tribometer equipment [11]. The scratch test was also evaluated using an acoustic emission sensor to confirm the penetration of the ceramic coating. Four different mechanical stress rates were used on the same material surface and the same load on AMSLER tribometer equipment (the rotating lower disc of the AMSLER machine is made of AISI 52100 bearing steel with a hardness of 62–65 HRc and a diameter of 59 mm both radially and axially). A tensometric data acquisition system was used to monitor the friction torque within the tribosystem. A Vishay P3 strain gauge bridge with four channels was employed with the specific soft program. The acquired data was processed by LabVIEW virtual instrument signal processing application. The mathematical relationships for friction torque and friction coefficient estimation and the LabVIEW program interface are presented in [11].

The electrochemical tests were achieved by linear potentiometry (potentiostat PGP 201) using a cell with three electrodes (1—the sample, 2—Pt electrode, 3—calomel saturated electrode). Before the experiment, the samples were cleaned in an ultrasonic bath using technical alcohol for 60 min [12]. The electrolytic solution used for testing was acid rainwater (mixing solution 1:1 of H_2_SO_4_ and HNO_3_ with pH = 3). These chemical substances are naturally present in the atmosphere; however, before industrialization, the advent of factories and the dependence on hydrocarbon (coal, gasoline etc.) acid rain was a rare event. In recent decades, acid rain has become more and more frequent, especially in the area of highly congested cities and heavily industrialized areas.

## 3. Experimental Results

### 3.1. Structural and Chemical Analysis of Ceramic Coatings

Deposition of the ceramic Al oxides (alumina) (two or four passes) using APS produce compact coatings, especially on the sandblasted samples, having an estimated thickness of 30 µm, respectively 60 µm, function almost linearly dependent on the number of passes [13].

**Figure 1:**
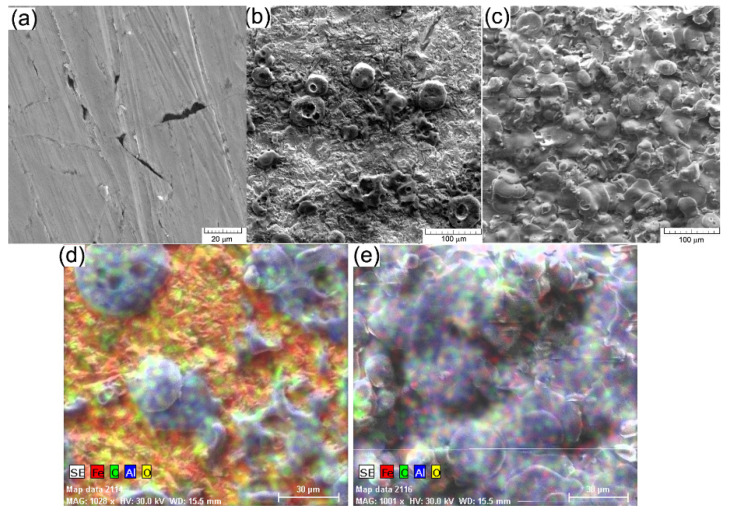
SEM images: (**a**) cast iron EN-GJL-250; (**b**) EN-GJL-250 + 2 layers of ceramic material; (**c**) EN-GJL-250 + 4 layers of ceramic material, in (**d**,**e**) there are shown distributions of elements inside the deposited ceramic coatings using two and four passes, respectively.

Further on, in Figure 1 there are shown, using the scanning—electron microscope, the surfaces of the coatings obtained by plasma spraying. From the viewpoint of the structure, the achieved coating on the grinded sample, but not sandblasted appears discontinuous, Figure 1d, with areas where the cast iron substrate can be seen. The discontinuities of the deposited coating, of any nature: pores, cracks, exfoliations etc. decrease the mechanical and chemical properties. For this reason, it is not recommended to achieve the plasma jet deposition without careful preparation of the surface at the macroscopic level to enhance the adhesion of the layer.

Figure 1c presents the morphology of the deposited coating on the sample blasted with glass (showing a higher roughness). On the surface, a compact layer can be seen, without discontinuities or exfoliations, showing local micro-cracks of micrometric sizes. The cracks do not penetrate the whole layer and occur mainly because of the temperature differences between the deposited layer and the colder substrate (layer achieved on the previous passing). The layer is obtained generally by zones with melt material that form a compact mass under the influence of the very high temperature, around 12,000 °C. The microstructure of the ceramic deposited coating of 60 μm, corresponding to four passes can be appreciated as having fewer cracks, fewer pores, and a more homogenous covering on the sample substrate with higher roughness. A higher roughness is an advantage for the adhesion properties of the ceramic coating because it provides greater anchor support.

For the chemical characterisation of thin layers achieved by thermal spraying on cast iron substrate, the distribution of chemical elements inside the deposited ceramic coating was analysed and the results are given in Figure 1d,e. The distribution was analysed for the following chemical elements: Al and O the components of the coating and Fe and C the elements of the substrate EN-GJL-250 cast iron. From the analysis of the morphology of the coating and the distribution of elements Al and Fe, it can be seen, in the case of the sample with two passes, a larger non-uniformity of the ceramic deposited coating. The area covered is a little more than 60% on the analysed surface, in this case, 0.18 mm^2^. The area chosen for analysis, Figure 1b is characteristic of the deposited layer and it shows the same aspect on the entire surface covered with alumina. The non-uniformities of the ceramic coating lead to total exfoliation of it during operation and the promotion of corrosion on the exposed zones to a solution of an electrolyte by comparison with the coated zone.

**Figure 2:**
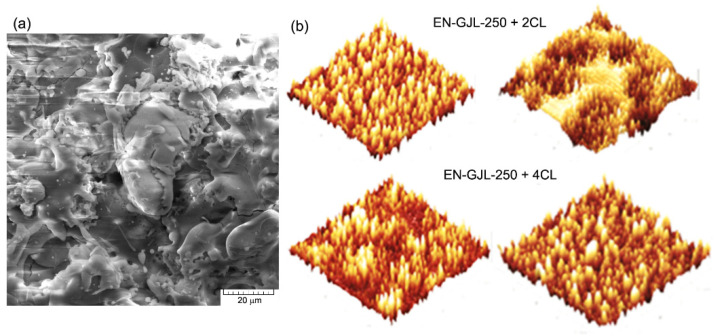
SEM images, in (**a**) and AFM, in (**b**) for the ceramic deposited coating.

The surface of the deposited ceramic coating was also analysed using an atomic force microscope (3D). On a surface of 64 µm^2^, Figure 2a, it can also be seen that for the case of four passes a more homogenous surface was obtained. After establishing a suitable sandblasting regime for the cast iron substrate there were achieved experimental samples with two and four passes of ceramic sprayed material (two and four ceramic layers deposited on the same sample).

**Figure 3:**
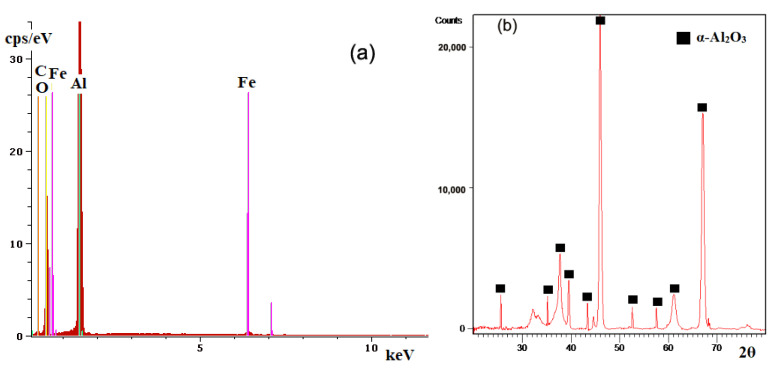
Chemical analysis of the ceramic coatings deposited on the metallic substrate. (**a**) EDS spectrum; (**b**) XRD spectrum.

Figure 3 shows the spectrum of energies characteristic of the chemical elements identified on the ceramic deposited coating on a cast iron substrate (the spectrum shown was identified for both experimental samples).

From the analysis of the spectrum in Figure 3a it can be seen the qualitative identification of elements of the ceramic coating, respectively, Al and O but also the elements Fe and C specific for the cast iron substrate. The elements characteristic of the substrate (Fe and C) were identified through the pores and cracks that occurred in the ceramic coating during the deposition process. The XRD spectrum, Figure 3b, identifies more characteristic picks for the phase α specific for alumina, the plot is from the sample with four layers. The qualitative phase analysis was performed using the PDXL2 (Rigaku) software and the database ICDD PDF4 + 2022. The qualitative phase analysis indicated the following polycrystalline phases: α-alumina and gamma-alumina (or eta-alumina) in very low percentages.

Table 1 shows in mass percentages (wt%) and atomic percentages (at%) the chemical composition of deposited ceramic coatings on cast iron substrate. In the case of the sample with two layers, it can be seen a large amount of Fe that signifies a lower coating percentage of the metallic material.

For the sample with four passes, the Fe content identified occurs because of the micro-cracks or of the pores existent in the ceramic coating or, of the low thickness of the layer in some areas, a fact depending on the quality of the deposition that can vary as a function of sample geometry. The high percentage of C for both cases is also due to the error of the EDS detector.

The structural and chemical analysis of the ceramic surface obtained after four passes during the deposition process shows a uniform surface of the coating with all areas of the substrate covered, Figure 1d. The distribution of Al and the lack of Fe signal confirm the fact that the metallic surface is completely covered after four passes of the plasma jet.

As a function of the application for which the coating is being deposited, it can be subject to some supplementary technical procedures of rolling or, heat treating for chemical and structural homogenisation, using a furnace or an acetylene flame. In this case, it is intended to maintain the roughness of the surface for increasing the coefficient of friction. For the braking discs, a self-rolling process occurs during the part operation.

### 3.2. The Analysis of the Behaviour of Tested Materials to Micro-Indentation

**Figure 4:**
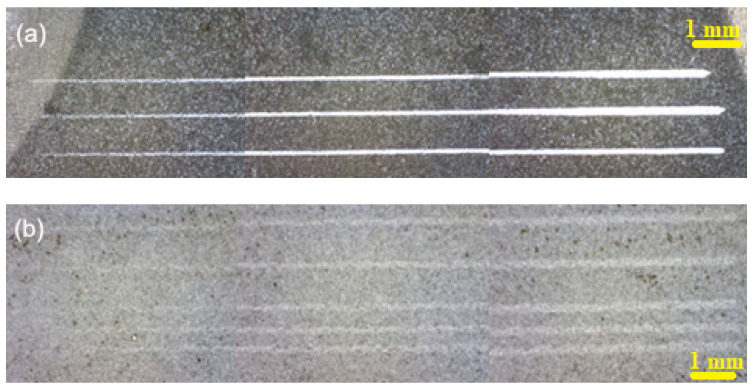
Identification and images of scratches using optical microscopy for the coatings: (**a**) two layers; (**b**) four layers.

For analysing the adhesion of the ceramic coatings to the metallic substrate and for determining the tribological properties, there were performed scratch tests on the experimental samples EN-GJL-250, EN-GJL-250 + 2 ceramic layers, and EN-GJL-250 + 4 ceramic layers. Figure 4 shows the general aspect of the scratches, from the starting point of the test, from the left side to the right side, and at the end of the test. The figures were made using 3 images obtained at the optical microscope, focusing on the areas that characterise the scratch from its beginning to its end. The scratch length achieved was 25 mm using a progressive load. There were several scratching tests aiming at the characterisation of the homogeneity of the tribologic properties of the superficial deposited coatings.

**Figure 5:**
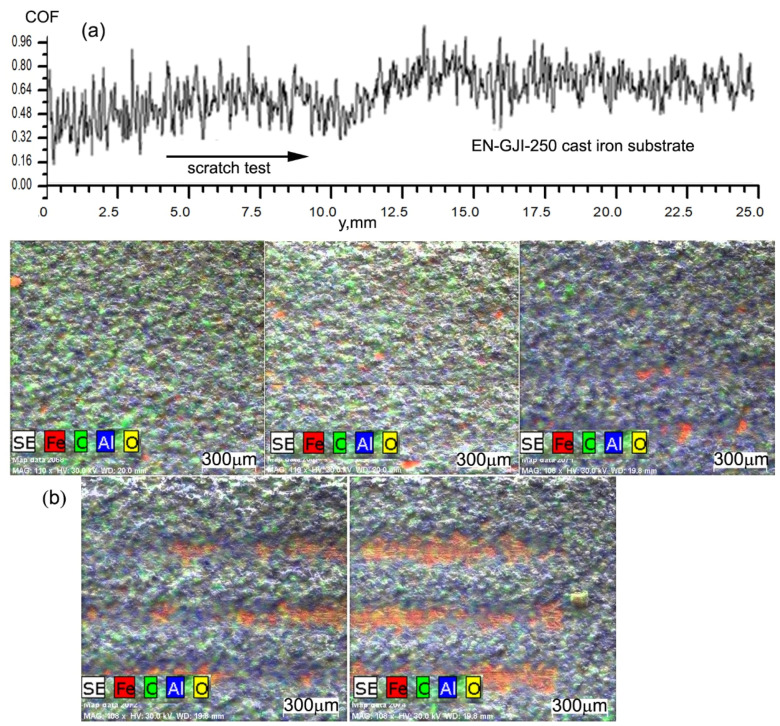
Analysis of the scratching test using the coefficient of friction behaviour: (**a**) variation of coefficient of friction on 25 mm distance; (**b**) distribution of elements Fe, C, Al and O on the surface of the scratched ceramic coating.

At the microscopic level, there were not seen exfoliations of the deposited ceramic coating and the uniform aspect of them shows good structural homogeneity of the layer. Figure 5 shows the characteristics of the scratching behaviour of the sample EN-GJL-250+4 ceramic layers. The scratching equipment was simultaneously operated with an acoustic sensor to record the initial behaviour of the ceramic coating as well as the penetrated layer together with the substrate after a period of time [14].

The experiment started with an initial load of zero Newton (0 N) (Fz) rising up to 8 N on a length of 25 mm. It can be seen in the evolution of the friction force and the acoustic emission a variation of the signals at 10.5 ÷ 11.5 mm, from the beginning of the scratch initiated on the ceramic surface, the area that probably represents the point when the metallic penetrator penetrated the ceramic coating. After this, the friction force increased due to the double effect of the stresses, one of the ceramic coating and the other one, of the EN-GJL-250 metallic substrate.

For the analysis of the behaviour of the coefficient of friction, Figure 4, extracted from the signal of the scratching test, the one obtained on the ceramic coating and after its penetration on the system ceramic coating—cast iron EN-GJI-250 is chemically analysed in certain zones. 

The coefficient of friction shows the same behaviour as the friction force, having a variation after 10.5 s from the start of the test, Figure 5a. The scratch obtained after the mechanical testing was analysed using electron microscopy SEM (on the areas from a to g) after characterising the mark at every 2 mm, Figure 5. Figure 5b shows the distribution of the elements Fe and C characterising the cast iron substrate and Al and O characterising the ceramic coating of Al_2_O_3_ on the areas (a), (b), (d), (f) and (g) exactly on the scratch mark. In the first two distributions from Figure 5b, there is no sign of penetration of the ceramic coating, this being evident in the (d) area by the significant increase in the element Fe signal on the scratch mark. The Fe signal is accompanied by the signal of the element carbon but not so obviously, due to its lower percentage. If in the (d) area, the ceramic coating was only partially penetrated in the following 10–14 mm it was gradually removed. The ceramic coating was removed completely in some areas, especially on the last stress portion. It can be seen in areas (f) and (g) from Figure 5b portions having the ceramic coating present on the scratch marks. Their presence can be explained by a superior adherence to the substrate in these areas or by settling the ceramic material under the force of scratching/pressing and its penetration into the metallic cast iron EN-GJL-250 substrate.

The microstructural analysis was performed starting with the final end of the scratch at each 2 mm until no variation of the ceramic coating microstructure was present, the area considered the starting point of the scratching test and which corresponds to the one obtained from calculus considering the total length of the scratch, respectively 25 mm. From a microstructural viewpoint, a chamfering of the ceramic coating can be seen, in Figure 5b, at 2 mm from the beginning of the scratching test, namely at a loading force of 1 ÷ 2 N, confirming the fact that the layers of Al_2_O_3_ are relatively soft among the ceramics but less brittle compared to very hard ones.

**Figure 6:**
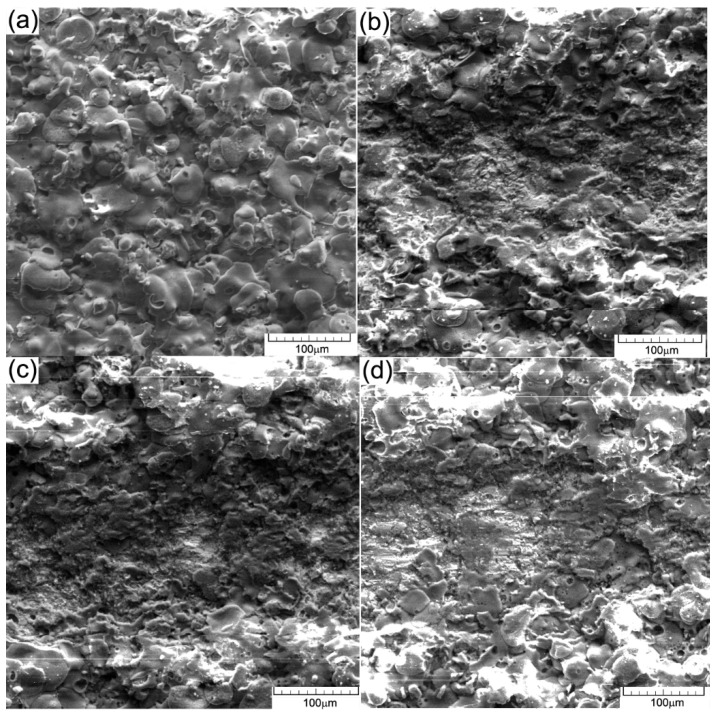
SEM images—details of the scratched areas on the ceramic coating (**a**) initiation of scratch, (**b**) first area of ceramic layer deformation, (**c**) local compaction of the ceramic layer and (**d**) end of the scratch mark.

The depth of the scratching marks without the penetration of the coating continues up to 10 ÷ 11 s of stress i.e., at a force of 4 ÷ 5 N. There cannot be seen areas with cracks on the edges of the scratch mark and nor in the areas of ceramic coating between scratches. The analysis at a higher magnification power, Figure 6a–d did not reveal the presence of the cracks or pores on the pressed ceramic surface, nor their appearance on the metallic substrate. 

The integrity of the ceramic coating is very little affected on the edges of the scratch mark, showing the high stability of the ceramic deposited layer. Inside the analysed scratch marks, they appear exfoliation areas of the ceramic coating, Figure 5b but also the presence of areas with a compressed ceramic layer. In practical applications where the increase in the wear coefficient is not especially aimed, additional processing of the coating, carried out mechanically or by heat treatment, is recommended, for the uniformity of the surface, the reduction of roughness and the homogenization of the coatings.

**Figure 7:**
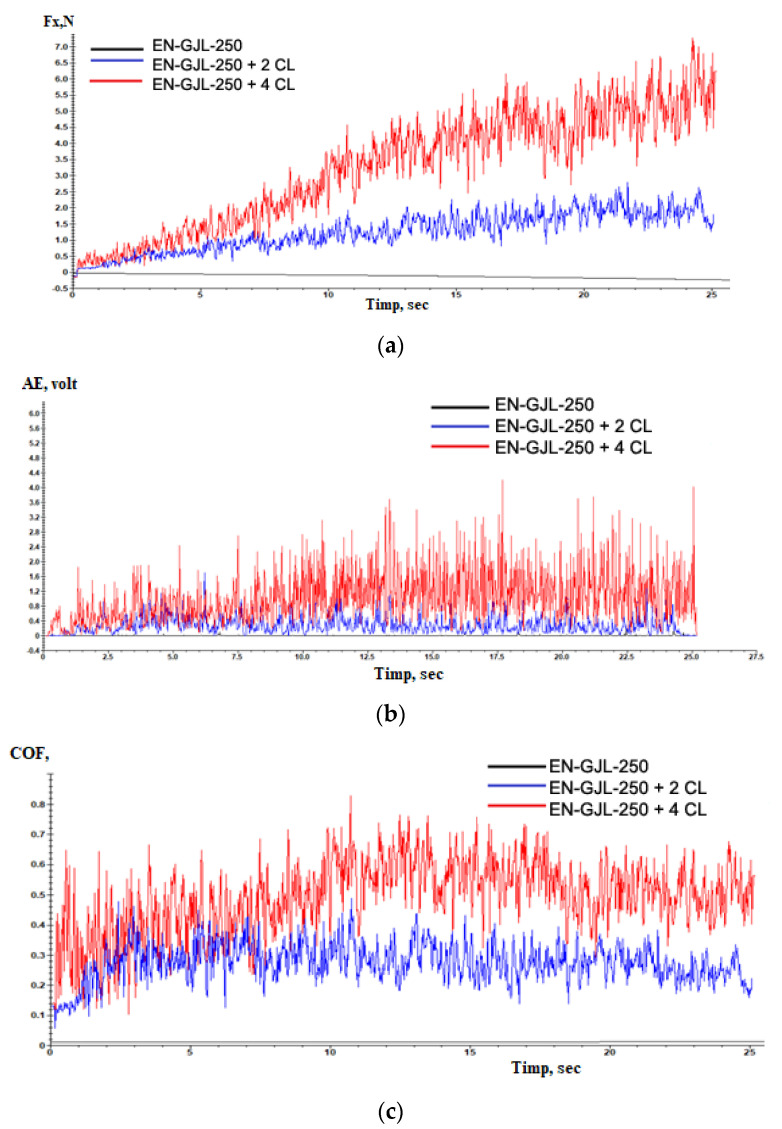
The behaviour of experimental materials EN-GJL-250, EN-GJL-250 + 2 ceramic layers and EN-GJL-250 + 4 ceramic layers at scratching: (**a**) friction force, (**b**) acoustic emission and (**c**) coefficient of friction.

Figure 7 shows the behaviour of experimental materials EN-GJL-250, EN-GJL-250 + 2 ceramic layers, and EN-GJL-250 + 4 ceramic layers for the variation: (a) friction force, (b) acoustic emission and (c) coefficient of friction during the scratching test.

From Figure 7a one can see that the friction forces are greater in the case of the samples with ceramic deposited layers by comparing with the friction force occurring on the EN-GJL-250 cast iron and which shows only slight variations in behaviour due to the differences in hardness between the metallic substrate characteristic of cast iron and graphite formations. In both cases of coatings (with two, respectively four layers) one can see an increase in the friction force after the penetration of the ceramic coating and the complex friction among the indenter on one hand and the ceramic coating and the substrate on the other hand. Additionally, it can be seen that increasing 2 ÷ 3 times the friction force in the case of the sample with the coating made by 4 ceramic layers compared with that having only two deposited ceramic layers as a coating.

In the case of acoustic emission (acoustic emission—AE), Figure 7b, the signal of the substrate is also an almost straight line compared with the emissions of the samples with ceramic coating.

Producing and developing cracks can be events having very short periods of time to occur and grow. The acoustic emissions were conceived for detecting the behaviour at fracturing and cracking of materials. Wakayama and Ishiwata [15] used AE detection to analyse and evaluate the ceramic micro-cracks, [16] for detecting the deterioration in composite ceramics and composites reinforced with fibres. The same authors used the AE detection method [17] to detect the part fracturing during the processing of the surface of engineered ceramics.

During the scratching tests, the AE technique was also used for monitoring fragile breaking [14]. The level of acoustic emissions is higher in the case of the sample with 4 ceramic layers, having a visible increase in the areas where the deposited coating was penetrated. The amplitude of the acoustic emission signal has increased significantly because of the severe vibrations of the indented that resulted from the initiation and propagation of cracks and/or during the removal of the material through plastic deformation of fragile breaking of the Al_2_O_3_ coating during scratching. In the case of a large fluctuation in the AE signal, as the amplitude is higher the damages caused to the deposited ceramic coating or substrate, in the case the substrate is reached, is more severe.

**Figure 8:**
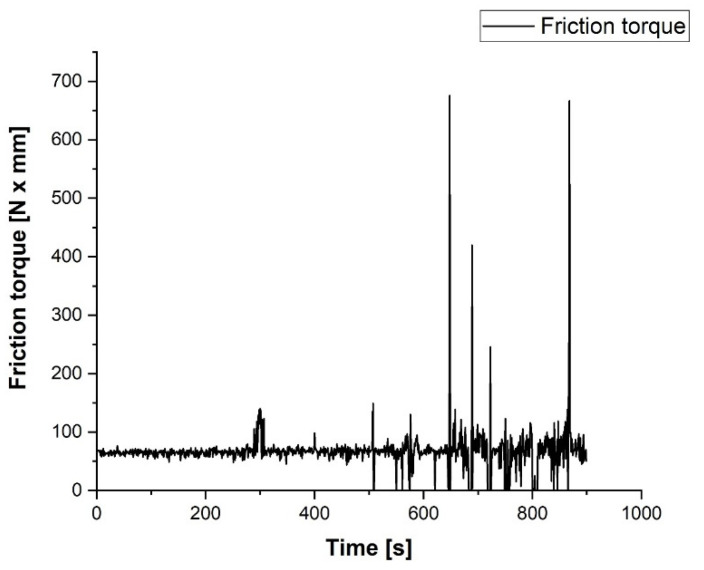
The friction test results performed on the AMSLER tribometer.

The coefficient of friction, Figure 8 shows a substantial increase in the case of the samples having ceramic coatings compared with the cast iron substrate. This increase is due to both the roughness of the ceramic layers and their nature. After the penetration of the ceramic coating, at the value of the coefficient of friction, it is added also the friction with the substrate that contributes in this manner to the identified increase in the coefficient of friction. The values of the coefficient of friction COF vary in a wide range because of the high resistance that the ceramic coating presents, determining a greater capacity for plastic deformation than the substrate. Despite these relatively high coefficients of friction —COF values, plasma-sprayed coatings are still accepted for many applications [18]. For applications that operate under severe wear conditions, these coatings may be supplemented by various additional treatments such as laser reshaping, sealing treatment, or surface grinding to improve the surface modification and therefore the coefficient of friction values [19,20]. If we consider the variation of the coefficient of friction of ceramic materials as coatings until their penetration (in the time range 9 ÷ 11 s) from 0 to 10 s of testing, one can see two zones of variation. Initially, the coefficient of friction grows suddenly up to values of 0.6 with a period of stability, after which it follows a slight decrease in the coefficient of friction down to 0.3 ÷ 0.4 probably because of the large degree of flatness of the deposited ceramic coating and the diminishing of roughness values of the coated surface. According to the model Czihos [21,22] the curves of the coefficient of friction–COF consist of three stages: initial wear, steady state and accelerated wear up to the point of penetration point and contact with the metallic substrate in this case. The third stage in the coefficient of friction—COF curves for the coated and worn samples in this study opposes the Czihos model. It seems that some tribological interaction due especially to the joint influence of the substrate and the edges of the ceramic coating in the penetrated zone affects the stabilization of the coefficient of friction—COF.

### 3.3. Wear Resistance Analysis

The coated sample was tested on an AMSLER apparatus using a disc made from ASTM 52100 bearing steel. The data collection was performed by a strain-meter that monitored the friction torque in the tribo-system. For data acquisition, it was coupled with a strain indicator and recorder Vishay P3 with 4 channels using its specific software. The acquired data were processed in Lab VIEW for virtual signal processing. The mathematical relations for estimating the friction torque and the coefficient of friction as well as the Lab VIEW interface are given in [23].

A friction test was performed on the AMSLER equipment at a rotation speed of 100 rpm and a constant axial load of about 60 N (6 kg). The evolution of the friction torque T_f_ in N x mm and of the coefficient of friction µ is shown in Figure 8. As one can see, in the first 5 min, the coefficient of friction between the coating layer and the ASTM 52100 steel disc was about 0.16 ÷ 0.18, the friction process, and wear being smooth and continuous. After 5 min, the coating layer was partially removed and the first metallic contact with a small surface raised the coefficient of friction up to 0.35, but just for seconds. The friction force of the contact became unstable, but within reasonable limits until the worn surface extended and most of the contact became metal on metal. 

After 500 s from the start of the test, the friction became dynamic and this can be explained by the intensification of some micro-seizure phenomenon on the metallic contact surface. In the last 5 min of testing, the dynamic phenomenon of sliding on the contact area of the tribological contact manifested itself by strong vibrations and collisions on the tested samples. Consequently, the variation in the coefficient of friction was very large. The test was stopped after 15 min due to the simple observation of the wear zone of the coated sample which revealed the complete removal of the coating layer in the contact area. The statistical analysis of the data acquisition process shows a value of the signal-to-noise ratio SNR = 1.67, which confirms the good quality of the acquired signal. For the last period of the test, the difficulty of the acquisition and the standard deviation have high values, confirming the fluctuation of data acquisition, the causes have already been mentioned. Comparing the results obtained with those previously reported for EN-GJL-250 one can see a coefficient of friction increased up to 0.17 for the entire test. These results recommend the coatings using Al_2_O_3_ for lighter-duty applications [24].

Future tests should be carried out in this direction, with a lower contact pressure and used as wear material instead of the steel disc, ferodou discs, or special materials used in braking systems.

**Figure 9:**
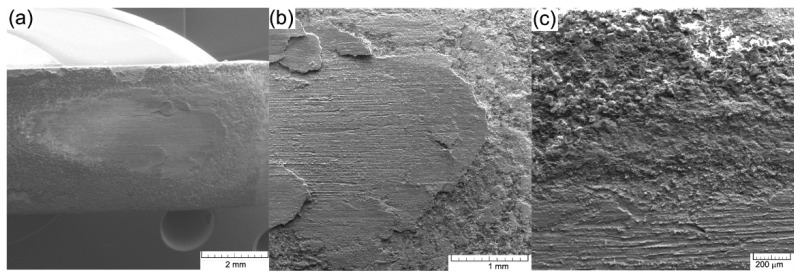
SEM images of the worn zone during the test: (**a**) wear trace; (**b**) detail of the wear end area; (**c**) wear edge.

**Figure 10:**
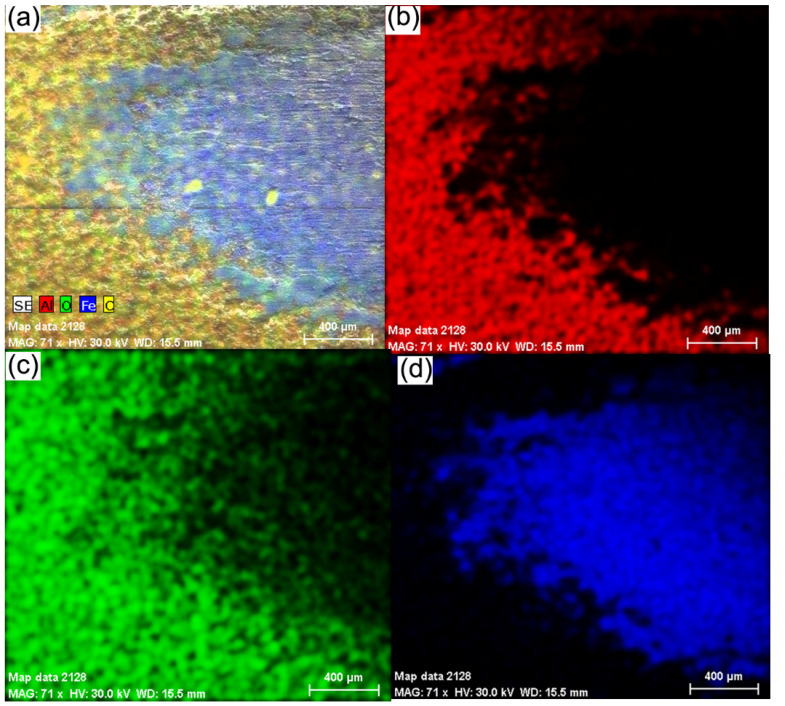
The chemical elements distribution for Al, O, F and C in the contact zone at the wear test: (**a**) distribution of all elements; (**b**) distribution of aluminium; (**c**) distribution of oxygen; (**d**) distribution of iron.

Figure 9 shows SEM images of the wear trace obtained after the test performed on the AMSLER equipment. The trace is about 4mm long and 2 mm wide. A complete removal of the ceramic coating from the contact area can be seen in Figure 10a.

The contact achieved during the experiment was extremely hard because it also engaged material from the substrate, Figure 9b. The ceramic material was subjected to advanced wear by being placed between two metallic materials, cast iron as a substrate and the steel disc as a wear material. The relatively brittle nature of the coating resulted in its exfoliation at the contact area but without further affecting the integrity of the coating near the wear trace, Figure 9c.

**Figure 11:**
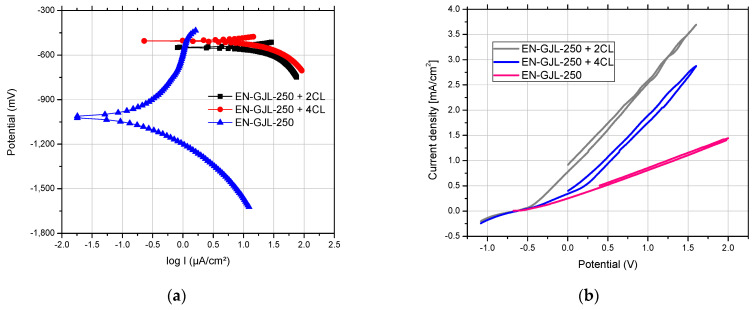
Potentiodynamic graphs for coated samples with Al_2_O_3_ of different thicknesses on EN-GJL-250 cast iron substrate compared with the free substrate EN-GJL-250 cast iron: (**a**) linear polarisation curves; (**b**) cyclic polarisation curves.

To highlight the wear zone in Figure 11, it is shown the distribution of Al, O, Fe and C elements in the contact zone of the wear test (a) distribution of all elements, (b) distribution of aluminium, (c) distribution of oxygen and (d) distribution of iron. 

Complete removal of the ceramic coating from the contact area can be seen. No compacted parts of the ceramic material were identified in the contact area. The ceramic coating shows exfoliations of micro-cracks and pores only at the beginning and end of the contact zone, the sides of the wear trace being unaffected by the test. The removal of the ceramic coating was achieved following strong mechanical shocks that primarily targeted the wear trace and less the surrounding zones that do not show affected surfaces.

### 3.4. Electro-Corrosion of Metal-Ceramic Systems

**Figure 12:**
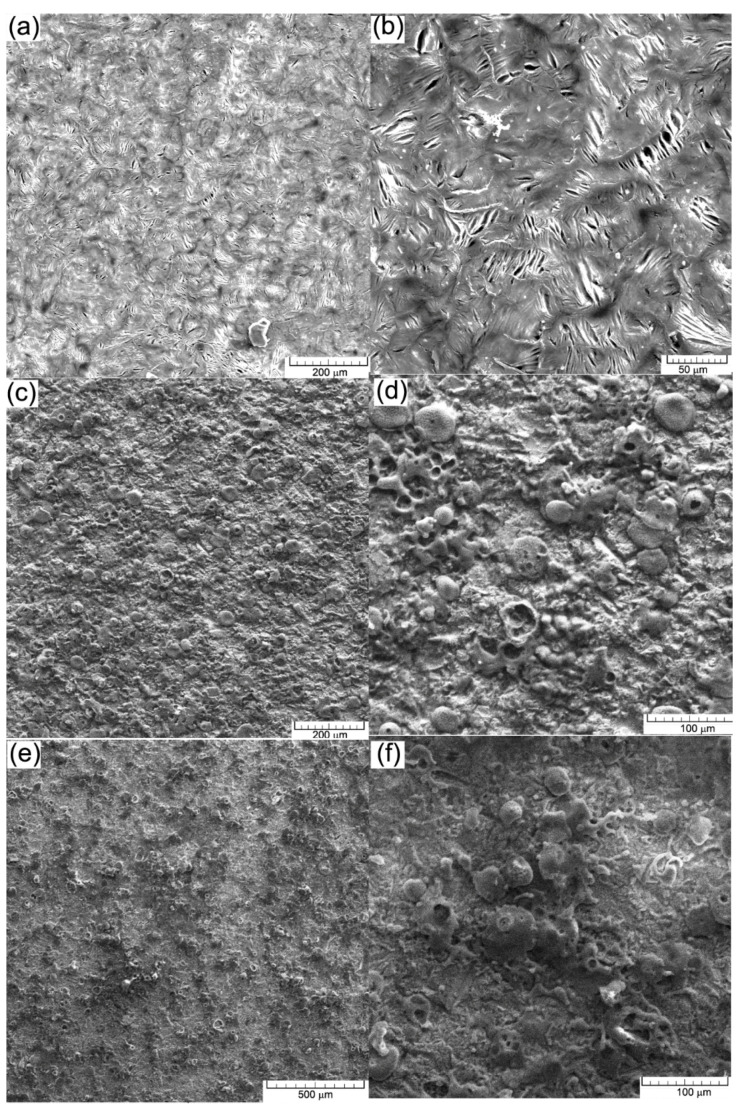
SEM images on the surface after the electrochemical test: (**a**,**b**) cast iron EN-GJL-250; (**c**,**d**) EN-GJL-250 + 2 ceramic layers; (**e**,**f**) EN-GJL-250 + 4 ceramic layers.

The experimental results show the electro-corrosion resistance of three samples (cast iron substrate EN-GJL-250, EN-GJL-250 + 2 layers of ceramic material Al_2_O_3_~30 μm and EN-GJL-250 + 4 layers of ceramic material Al_2_O_3_~60 μm) in electrolytic acid rain solution. Figure 11 shows the linear potentiodynamic graphs for Al_2_O_3_ coatings of different thicknesses on EN-GJL-250 cast iron compared with the substrate EN-GJL-250 cast iron and in Figure 12b the cyclic polarisation curves. The liner potentiodynamic graphs were represented in the range of potential: −0,8 ÷ 1 V using a scanning speed of 1 mV/s [25]. The corrosion speed can be correlated with the corrosion current intensity or the current density based on the law of Faraday [26]. For the experimental cases corrosion speeds of the order of millimetres per year for the cast iron EN-GJL-250 and micrometres per year for the coated metallic materials. From Figure 11a one can see a big difference in behaviour between the cast iron material and the cast iron coated with ceramic layer material. No significant difference was noticed for the curves of cyclic polarisation, Figure 12b. The samples coated with ceramic layers show similar behaviour with an almost non-existent anodic reaction.

The cathodic curve of cyclic graphs, Figure 11b, shows a similar trajectory to the anodic curve—having a reduced hysteresis loop, and the current density in the passive region is similar to that registered during direct scanning (anodic) at the same potential [12]. The slight difference between the anodic line and the cathodic line (the lack of a loop) is related to the stability of the surface and the competition between diffusion and dissolution in the case of pitting corrosion. The pitting corrosion occurs based on a very fast diffusion process having a semi-circle dimension appearance. In the first part of the cathodic process (the reversal line), the effects of the dissolution process are reduced and the time for the continuation of the diffusion is limited and usually insufficient.

The main parameters of the corrosion process (E_0_ and j_cor_) obtained by processing the linear polarization graphs are centralized in Table 2. The corrosion current thus determined is, in fact, the corrosion current that occurs at the metal/corrosive environment interface when the metal is introduced into the solution and cannot be directly measured by electrochemical methods. The open circuit potential—(OCP) shows large differences between the EN-GJL-250 cast iron and the metallic material coated with ceramic layers due to the influence of the inert material layer on the corrosion resistance of the entire assembly. The bias resistance validated the OCP values and the corrosion current values.

The corrosion current of the starting material (EN-GJL-250) is four to five times greater compared with the value recorded for the ceramic coating samples. The corrosion rate was 30 to 40 times higher for EN-GJL-250 compared to coated samples.

Scanning electron microscopy (SEM) (VegaTescan LMH II) was applied for the morphologic analyse of coatings and structure of the substrate material before the electrochemical tests. The results obtained are given in Figure 12a. In Figure 12b,c, the micrographs of the coatings show a dense microstructure with high cohesion and small surface cracks. Furthermore, some areas with pores can be seen in both coated samples. During the deposition process, micro-cracks and pores gather forming larger cracks. The main reason for the occurrence of these defects is the very short solidification time of the material in the atmosphere and the temperature difference between the deposited layers, the phenomena of dilation/constriction involving very important thermal stresses.

The coated surfaces in both cases show a complete melted zone. Further on, some formations of solidified material can be seen, but for these, solidification occurred later than in the underlying layers. A relative degree of homogeneity of the coating layer is crucial for the increase of the corrosion resistance of the substrate.

Figure 12 shows SEM images of the experimental materials after the electrochemical tests (a) and (b) cast iron EN-GJL-250, (c) and (d) EN-GJL-250 + 2 ceramic layers and (e) and (f) EN-GJL-250 + 4 ceramic layers at two different magnifications 200× and 1000× respectively.

In all cases, shown in Figure 12, the general corrosion detected on the cyclic polarization curves is confirmed, Figure 12b, without specific areas of corrosion (pitting). Some coatings from Figure 12d,f show, especially on the areas represented by the ceramic material, pitting corrosion at the outer part of the particles. This behaviour does not represent the entire surface, being localised only zonally and not being recorded by the potentiostat in the cyclic curves [27]. It can thus be stated that micro-zonally the larger agglomerations on the surface of the coating morphologically manifest a behaviour specific for pitting corrosion. If the environment continues to be aggressive (the dissolution rate is high enough to overcome diffusion) the pitting pits appearing at the surface of the ceramic coating can penetrate through the ceramic layer and the electrolyte will come into contact with the metallic substrate, which is much more susceptible to corrosion.

In the case of samples coated with ceramic materials, an aggressive surface attack can be seen even though the resistance of the outer oxide layer contributes a very high corrosion resistance, oxide being a material with good chemical inertia. Normally, the inert behaviour of ceramic materials that protect the substrate (such as alumina) should keep the surface intact. The pores and the micro-cracks present in the coatings became larger after the electro-corrosion tests because the original micro-pores and micro-cracks were damaged and chemically attacked. The main reason for the corrosion is given by the initial existence of pores and cracks in the coated surfaces. The SEM images shown in Figure 12d,f suggest that the corrosion damage was mainly confined to the coating defects (i.e., pores and cracks) [27,28]. It can be seen that some spherical corrosion products have formed around the coating defects. The results of the EDS analysis, Table 3 indicated that the corrosion products were mainly composed of Fe and O. It was shown that electrochemical corrosion produced compounds on the cast iron substrate during the electrochemical experiments.

The corrosion process occurs mainly through the cracks and pores in the ceramic layer that allow the contact of the electrolytic solution with the metallic substrate. In all three cases, the materials show a pronounced oxidation on the surface, especially on the cast iron EN-GJL-250. This is because a part of the oxygen, in the other two experimental cases, is part of the coating and just a percentage of it participates in the formation of oxides. Generally, the ceramic layer was penetrated by the electrolyte to the substrate because iron oxides appear at the surface. Since the ceramic top layer and the metallic bonding layer are very passive, there is not much difference in their electrical potential and no electrical micro-piles formed between the two materials.

## 4. Conclusions

After analysing the experimental results, the following conclusions can be drawn:

- Microstructurally a chamfering of the ceramic coating can be seen starting at 2 mm from the start of the scratch test i.e., at a force of about 1 ÷ 2 N, which confirmed the fact that the Al_2_O_3_ coatings are relatively soft compared with other ceramics but less brittle than the very hard ones;
- No area with macro-cracks was noticed on the edges of the scratch marks and nor in the ceramic material between scratches. Analysis at a higher magnification of the surface image did not reveal cracks or pores on the pressed ceramic surface, nor did they appear in the metallic substrate;
- We observed that the friction forces are higher in the case of the coated samples compared with the friction force specific for the cast iron EN-GJL-250 and which shows only slight variations in behaviour due to hardness differences between the metallic matrix and the graphite formations characteristic for cast iron. In both cases of the coatings (with two deposited ceramic layers, respectively four ceramic layers) one can see an increase in the friction force after the penetration of the ceramic coating and the complex friction between the indenter on one hand and the ceramic penetrated layer and substrate on the other hand. Furthermore, a 2 ÷ 3 times increase in the friction force can be seen in the case of the sample coated with four ceramic layers compared to the one coated with two ceramic layers;
- In the case of experimental samples with deposited ceramic coatings, the general corrosion detected on the cyclic polarization curves was confirmed, without specific areas of corrosion (pitting). Some coatings show, especially on the areas represented by the ceramic material, pitting corrosion at the outer part of the particles. This behaviour does not represent the entire surface, being localised only zonally and not being recorded by the potentiostat in the cyclic curves. It can be stated that zonally the larger agglomerations on the surface of the coating morphologically manifest a behaviour specific for pitting corrosion. If the environment continues to be aggressive (the dissolution rate is high enough to overcome diffusion) the pitting pits appearing at the surface of the ceramic coating can penetrate through the ceramic layer and the electrolyte will come into contact with the metallic substrate which is much more susceptible to corrosion. The corrosion current of the starting material (cast iron EN-GJL-250) is four to five times greater compared with the value recorded for the ceramic coating samples. The corrosion rate is 30 to 40 times higher for cast iron EN-GJL-250 compared to coated samples.

## Figures and Tables

**Table 1:** Chemical composition of ceramic coatings deposited on cast iron substrate.

Element/Sample	Al		O		Fe		C	
	wt%	at%	wt%	at%	wt%	at%	wt%	at%
Powders Al_2_O_3_	54.71	41.73	45.29	58.27	-	-	-	-
EN-GJL-250 + 2 ceramic layers	40.83	32.72	38.01	49.37	16.5	9.7	4.67	8.21
EN-GJL-250 + 4 ceramic layers	50.93	38.11	43.78	55.25	1.7	0.6	3.58	6.02
Error EDS %	1.7		1.1		0.2		0.1	

Standard deviation (SD, from 20 determinations): Al: ± 1.2; O: ± 0.9; Fe: ± 0.1; C: ± 0.2.

**Table 2:** Electrochemical parameters after the corrosion tests in acid rain solution.

Sample	OCP mV	E_0_ mV	b_a_ mV	b_c_ mV	R_p_ ohm.cm^2^	J_cor_ µA/cm^2^	V_cor_ mm/Year
EN-GJL-250 + 2 ceramic layers	−488	551.1	-	−468.8	1448	30.01	0.11
EN-GJL-250 + 4 ceramic layers	−430	505.2	-	−337.6	1967	26.15	0.09
EN-GJL-250	−716	−1016.8	659.8	−347.9	324.3	138.3	3.59

**Table 3:** Chemical composition of the experimental materials after the electro-corrosion [27].

Element/Sample	Fe		O		Al		C		Si	
	wt%	at%	wt%	at%	wt%	at%	wt%	at%	wt%	at%
EN-GJL-250	49.01	22.39	42.13	65.13	-	-	3.4	6.9	3.9	3.7
EN-GJL-250 + 2 layers	31.98	14.7	37.16	49.52	23.93	18.75	4.53	10.85	2.33	1.78
EN-GJL-250 + 4 layers	34.09	16.77	30.67	45.73	26.93	27.58	4.86	11.12	3.3	3.2
EDS Error %	0.65		1.0		0.5		0.8		0.1	

SD: Al: ± 1.2; O: ± 0.9; Fe: ± 0.1; C: ± 0.2, Si ± 0.1.

## Data Availability

Not applicable.
